# Stakeholders’ experiences with a clinician-led access programme linking evidence generation and reimbursement for precision cancer treatments: the drug access protocol in the Netherlands

**DOI:** 10.2340/1651-226X.2026.45000

**Published:** 2026-01-14

**Authors:** Christine Leopold, Atse H. Huisman, Kevin J. G. M. Vlaar, Haiko J. Bloemendal, Sahar Barjesteh van Waalwijk van Doorn-Khosrovani

**Affiliations:** a Division of Pharmacoepidemiology and Clinical Pharmacology, Utrecht Institute for Pharmaceutical Sciences (UIPS), Utrecht University, Utrecht, The Netherlands; b Department of Medical Oncology, Leiden University Medical Centre, Leiden, The Netherlands; c Health Insurers the Netherlands, Zeist, The Netherlands; d Department of Medical Oncology, Radboud Institute for Health Sciences, Radboud University Medical Center, Nijmegen, The Netherlands; e CZ Health Insurance, Tilburg, The Netherlands

**Keywords:** Health technology assessment, reimbursement mechanism, medical oncology

## Abstract

**Background and purpose:**

In the current landscape of tumour-agnostic oncology drugs receiving European Medicines Agency (EMA) authorisation, Health Technology Assessment (HTA) bodies face challenges in assessing these innovative drugs. Due to these products’ non-randomised, single-arm nature, uncertainty exists regarding their real-world benefit. In the Netherlands, the Drug Access Protocol (DAP), a programme developed by oncologists, insurers and the healthcare public institute, aims to provide an innovative solution to address this uncertainty. This study aims to investigate the key characteristics, enablers and challenges of the programme by exploring stakeholders’ perceptions.

**Patient/material and methods:**

A qualitative, semi-structured interview study was conducted. A supporting interview guide was drafted using available literature and a flowchart figure to illustrate the process. Interviews were conducted with market authorisation holders (MAHs) who participated in the programme, the insurer, the DAP study management and the DAP’s governance committee. Recorded interviews were transcribed, pseudonymised and subsequently coded using NVivo software. Inductive thematic analysis was used to identify common themes, enablers and challenges for participating in the programme.

**Results:**

In total, eight organisations were interviewed. Although MAHs indicated several enablers (e.g. providing patient access, collecting real-world data), several challenges (e.g. the lack of transparency) lead to questions regarding the feasibility of the programme. Health insurers acknowledge these outcomes and expect products that obtain regular reimbursement to serve as an example.

**Interpretation:**

As the Drug Access Protocol may be a promising solution to mitigate uncertainties for healthcare decision-makers, implementation challenges can hamper its feasibility. Addressing these challenges could realise the potential of such programmes.

## Introduction

In recent years, increased spending on oncology medicines is a rising problem of global concern as well as in the Netherlands [1].

This is mostly due to the increasing number of approvals of new oncology medicines combined with rising prices for new medicines [2–4]. At the same time, for many new innovations, there is uncertainty about their clinical effectiveness, especially when evidence comes from non-randomised, single-arm trial designs [5]. This uncertainty challenges current health technology assessment (HTA) processes including the assessment of a product’s cost- and clinical effectiveness as well as reimbursement negotiations, often leading to delays in reimbursement [6, 7].

To overcome these HTA and pricing challenges, there has been a shift towards outcome-based managed entry agreements (OB-MEAs), which aim to provide earlier access to medicines while collecting real-world evidence (RWE) on the clinical effectiveness [8–10].

In the Netherlands, an innovative early access scheme (a coverage with evidence collection programme) linking evidence generation and reimbursement for anti-cancer drugs, the so-called Drug Access Protocol (DAP), was introduced in February 2021 by the Dutch Health Insurer umbrella organisation (Zorgverzekeraars Nederland, ZN, from hereon after referred to as payer) together with the Dutch Association for Medical Oncology (NVMO) and the Netherlands Cancer Institute (NKI). The DAP is a prospective, open-label, non-randomised protocol that collects real-world efficacy and safety data. The original purpose of the DAP was to create a platform that provides an overview of all Compassionate Use Programs and Named Patient Programs in oncology in the Netherlands to provide equal participation opportunity for patients and facilitating data collection when needed. However, the platform quickly developed a parallel reimbursement programme, managed by the payer, to provide faster, controlled and coordinated access to cancer therapies (including potentially off-label indications) awaiting reimbursement in the Netherlands. Simultaneously, the programme mitigates the risk of adopting therapies with uncertain effectiveness by collecting prospective real-world data (RWD) on the efficacy and safety of these drugs [11–15]. A drug for a certain indication can only be part of the DAP if it meets the clinical relevance boundaries, specifically for non-randomised controlled trials for solid tumours: the PASKWIL-NRS (Table 1) [16–23].

**Table 1:** Products incorporated in the DAP programme, as of 27th January, 2025.

Drug	Indication	EMA authorisation status	DAP admission	Reimbursed*
Cemiplimab	Locally advanced or metastatic cutaneous squamous cell carcinoma (laCSCC; mCSCC)	CA on June 2019, Standard MA as of July 2022	February 2021	Regular, as of January 2024
Larotrectinib	Adults and paediatric patients with locally advanced or metastatic solid tumours expressing NTRK gene fusion and who have no satisfactory reaction to standard treatment(s) or where no standard treatment exists, or is indicated	CA as of September 2019	October 2021, No contract ZN, through VT trajectory	Regular, as of September 2023
Entrectinib	Adults and paediatric patients (aged 12 years and older) with locally advanced or metastatic solid tumours expressing NTRK gene fusion and who have no satisfactory reaction to standard treatment(s) or where no standard treatment exists, or is indicated	CA as of July 2020	October 2021, No contract ZN	Regular, as of September 2023
Capmatinib	Locally advanced or metastatic NSCLC for which standard anti-cancer treatment is no longer available or indicated. Only MET exon 14 skipping mutations, second-line treatment after immunotherapy and/or platinum-based chemotherapy	Standard MA as of June 2022	NPP June 2020, No contract ZN	No, inclusion stopped in 2022 due to the end of MAH collaboration
Selpercatinib	Advanced RET-fusion positive non-small cell lung cancer	CA as of February 2021	May 2022	Regular reimbursment upon completion of the DAP period and based on available randomised data in the first-line setting, July 2024
Selpercatinib	Advanced RET-mutant medullary thyroid cancer	CA as of February 2021	May 2022	Regular reimbursment upon completion of the DAP period and based on available randomised data in the first-line setting, July 2024
Selpercatinib	Advanced RET fusion-positive solid tumours, when treatment options not targeting RET provide limited clinical benefit or have been exhausted	CA as of April 2024	March 2025	Recuiting
Tepotinib	Advanced NSCLC harbouring MET exon 14 skipping mutations, who require systemic therapy following prior treatment with immunotherapy and/or platinum-based chemotherapy	Standard MA as of February 2022	July 2022	Regular, as of July 2025
Amivantamab	Locally advanced or metastatic NSCLC with EGFR exon 20 insertion mutation, requiring systemic therapy after platinum-based chemotherapy	CA as of December 2021	November 2022	No, currently in the follow-up phase (expected results by end of 2025)

CA: Conditional Authorisation; MA: (Standard) Market Authorisation; MAH: Marketing authorisation holder; VT: Voorwaardelijke Toegang (OB-MEA programme by Dutch HTA body together with Dutch Ministry of Health); NPP: Named Patient Programme.

* = Reimbursement status specific for the indication initiated in DAP.

To date, no research has been conducted taking a health system’s perspective on the DAP process, including stakeholders’ experiences. Hence, the aim of this study is to describe the key characteristics, benefits and challenges of the DAP, as a unique example of a bottom-up collaborative initiative, with a novel OB-MEA, and to explore stakeholders’ experiences with the programme.

## Patients/material and methods

This qualitative study consisted of in-depth semi-structured 60-min interviews that took place via Microsoft Teams between the period of 28th November 2024 and 24th of February 2025. For the interviews all relevant stakeholders including representatives from the Dutch payer, the DAP study management, as well as the DAP’s governance committee, and all involved marketing authorisation holders who have participated in the DAP programme were invited to participate. Participants recruitment was coordinated via the co-authorship team.

**Figure 1:**
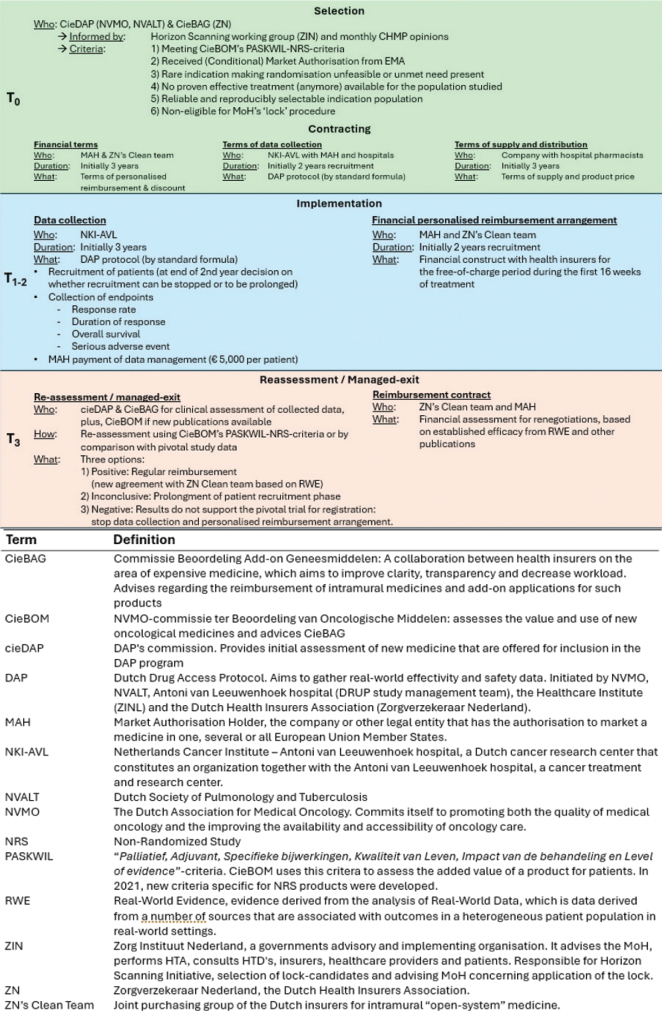
Flowchart illustrating the DAP process.

The interview guide consisted of a flowchart of the DAP process (see Figure 1) as well as of open-ended questions. Questions were divided into four topics: the DAP process, financial agreement, data collection and overall thoughts (see Annex A). The interview guide and the DAP process flowchart were shared with participants prior to the interview. During the interviews, participants were invited to use the flowchart as a starting point to share their experiences on the DAP process. In one case, a participant shared additional written responses on the interview guide, which was included in the data. The interviews were video recorded, and the audio was automatically transcribed using Amberscript, followed by manual editing to ‘intelligent verbatim’ transcript (removing speech disfluencies such as ‘um’, filler words such as ‘you know’ and sentence re-starts) and pseudonymisation. Transcripts were shared with participants upon request. All pseudonymised transcripts were coded using NVivo software (version 15). Thematic analysis was conducted guided by Braun and Clarke’s approach [24], with a focus on enablers and challenges that emerged from the participants’ experiences with the DAP programme. Transcripts were re-read by one of the researchers (KV) for a comprehensive understanding of the data. Themes were generated using an inductive thematic analysis approach. . From these common themes, enablers and challenges emerged that are further described in the results section.

Additionally, the DAP process flowchart was adjusted based on the feedback received during the interviews.

## Results

In total eight organisations agreed to participate in the interview (with mostly two experts per organisation); the participants represent five MAHs of the products enrolled in the programme (two products were in the early phase and therefore excluded from recruitment), the Dutch payer, the DAP’s governance committee and the DAP’s study management team.

As shown in Table 1, seven products are included in the DAP programme, comprising nine indications (selpercatinib enrolled four indications), and Figure 1 illustrates the different phases and timelines of the DAP process.

### DAP process

#### The initiation phase (T_0_)

T_0_ aims at selecting eligible products, which is done by the Dutch Association for Medical Oncology , the Dutch Society of Pulmonology and Tuberculosis and the payer. This process is informed a.o. by the half-yearly results of the Dutch HTA body’s Horizon Scan, which provides an overview of medicines expected to come into the market in the upcoming 2 years [25]. For a product to be eligible, it must meet the minimally clinically important difference defined by the Dutch Association for Medical Oncology for indications supported by (a) non-randomised trial(s). These criteria are referred to as the PASKWIL-NRS criteria (Table 2). As evidence from a non-randomised trial is not considered sufficient for regular reimbursement, inclusion in the DAP is mandatory for reimbursement.

**Table 2:** PASKWIL Criteria for Non-Randomised Studies: a product must meet all the requirements listed below to receive a positive opinion.

Requirements	Description
**1**	The indication for which the treatment is registered is rare.
**2**	In the related indicated area, either no treatment options with proven clinical benefit exist or all available treatments have been exhausted
**3**	The patient population can be selected reliably and reproducibly.
**4**	Preferably, there is a biological rationale for the mechanism of action of the treatment.
**5**	When assessing clinical value based on the objective response rate (ORR), a combined criterion is used involving the lower bound of the 95% confidence interval (95% CI) of the ORR and the point estimate of the median duration of response (DoR). The treatment is considered clinically relevant and valuable for the target population if: ORR > 40% **and** DoR > 4 months, **or** ORR > 30–40% **and** DoR > 8 months, **or** ORR > 20–30% **and** DoR > 12 months.
**6**	In a non-randomised study, overall survival (OS) or progression-free survival (PFS) is clinically meaningful if the gain in OS or PFS compared with the implicit or explicit control is more than 16 weeks (lower bound 95% CI).

CI = confidence interval; ORR = Objective Response Rate; DoR = duration of response; OS = overall survival; PFS = progression-free survival

Therefore, once a product is selected for a specific indication, negotiations and contracting begin. During the contracting phase (also part of T_0_), the MAH negotiates contracts with relevant stakeholders concerning financial terms (with payers), data collection terms (with the Netherlands Cancer Institute / Antoni van Leeuwenhoek hospital) and supply and distribution terms with the selected hospitals. These hospitals are selected by payers, as they meet the requirements to provide care for these rare indications. Contracts are usually signed for a 3-year period.

#### The implementation phase (T_1-2_)

After agreement on all terms is reached, the implementation phase is initiated (T_1-2_). Throughout this phase, the drug is reimbursed based on a personalised reimbursement scheme, with potential additional arrangements between the MAH and payers. Recruitment usually takes 2 years. Data are gathered by a master protocol for each product and a standard electronic Case Report Form (eCRF) for each patient. Sometimes, a product-specific section is added to the protocol, which, for instance, may be relevant for side effect management. In all hospitals, data are monitored, managed and analysed by The Netherlands Cancer Institute / Antoni van Leeuwenhoek hospital , a primary cancer institute for research and care. During the data gathering period, regular updates regarding the number of patients are provided to all stakeholders. Just before the end of the second year, DAP’s governance committee and the payer’s’ Add-on Drugs Assessment Committee assess whether the number of patients recruited and all other publications on the product and indication are expected to deliver the needed level of evidence for a re-assessment or the recruitment phase should be prolonged. When the required number of patients enrolled has been met and/or there are additional evidence available from other studies or registries, recruitment ends after a maximum of 2 years.

#### Follow-up and the re-assessment phase (T_3_)

When recruitment stops, the follow-up period of 6–8 months begins to ensure sufficient follow-up for the last patients recruited. After this, the re-assessment phase (T_3_) starts. All new patients who require treatment in this third year will be treated according to the protocol but outside the DAP. This means that no data will be collected for these new patients. Reimbursement, however, will continue to follow the personalised reimbursement scheme and any arrangements between the MAH and payers. This provision is intended to prevent any gap in access during the follow-up and re-assessment phase. The product is re-assessed to make sure that the additional evidence available supports the original clinical trial results. This means that the results of DAP should not differ significantly from the data of the pivotal trial. Because the number of patients may be limited, the final decision considers not only DAP data but also all other published RWE and relevant publications. . If the re-assessment results in a positive outcome, renegotiations with the payer start on the reimbursed price of the product based on the DAP data. DAP data guide the financial arrangements after the DAP so that the drug’s regular reimbursement reflects its actual value.

### Enablers of participating in the DAP programme

#### Providing earlier access to patients

Table 3 summarises the main aspects that experts perceive as an enabler or challenge in their participation in the DAP programme.

**Table 3:** Enablers and challenges experienced by participants, concerning participating in the DAP programme.

Enablers	Quotes
Providing earlier access to patients	*‘[…] From a pragmatic perspective, we were happy that the DAP was there. We were happy to join. We could get access to patients, and patients get access to this drug two and a half years sooner than in France, for example, where they didn’t have such a programme. So we were happy.’ [P1]*
Mitigating uncertainty on cost-effectiveness	*‘[…] I think the drug access protocol can help us in looking at routes which are beyond the EMA and looking at ways to address uncertainties as well, but also making sure that innovations can really provide value for those patients who need it.’ [P3]*
Clear timeframes and roles of stakeholders	*‘No. What I would like to do is mention one very positive thing that is the DAP loket [english translation: counter], so the ticket window, the DAP loket. That’s a team that’s based in the NKI and they do the data collection, they do the inclusion of the patients. They are very responsive. They are proactive. They send us reports, aggregate reports of the inclusion of the patients, start-stop dates and like that. So that’s a very positive thing.’ [P1]* ‘*There’s nobody really owning any of this, and taking the lead in any of this. We’re continuously chasing people to follow these timelines and stop recruitment. We are trying to stop them from recruiting new patients, because this is the evaluation year and the follow up year that we’re in right now. We’re sharing with cieDAP that they should plan their analysis, that it’s time now. So, these [cieDAP] are doctors that have practices and a lot of things on their minds. They’re not really owning it and managing it.*’ *[P1]* ‘*And after [three years of data collection], you don’t know what the negotiation will be […]. And when the CieBAG assesses the drug and assesses in a positive way. So the state of practice and science has been established, you don’t know upfront what they will do. You can imagine sometimes what they will do, but you don’t know what will happen. You can be sent to the Clean Team to make a price deal, or not. You don’t know upfront.’ [P1]*
Real-world effectiveness in the Netherlands	*‘So if the conclusion is that we don’t have the time [to wait for EMA required follow-up data], or that it takes too much time, then perhaps we need to decide, yes we go on and we collect this real world data in the Netherlands because patients are waiting, it’s too promising, we want to go further with it. And then you can decide that it takes too long and we don’t wait, for example.’ [P4]*

Challenges	Quotes

Financial costs of data collection	*‘[…], [assessing the fair market value] took a very long time because they couldn’t really provide sufficient arguments for why there should be €X and to provide that this was a fair market value. And I think that is still the issue, also if you want to enter the DAP as a company, that global offices think this is illegal.’ [P2]*
Rigidity of the protocol	*‘[…] what we experienced is also there is no flexibility in the drug access scheme, how it’s set up. So the 16 weeks is fixed. And if you think about the uncertainty and then also the financial conditions linked to such a time point, those are not fit for purpose for every therapeutic area or every drug. And that makes it also more difficult I think, to say if you would agree to such a scheme […]. Because that’s a very rigid protocol, which is one size fits or should be a one size fits all, which is actually not the case.’ [P5]*
Lack of transparency for MAHs	*‘[…] There was no target of patient numbers mentioned in advance. […] In the first two years, we were unclear about what would be the criteria for the drug access protocol to work with. […] That’s something where we didn’t feel on par with regards to information levels.’ [P3]*
Data sharing	*‘[…] we would have wanted to be updated on a more regular basis. Also about the number of patients included, but also the number of patients who continued after […] [the free-of-charge phase]. […] you don’t know how many patients after four months will continue the treatment. […] I think it would be fair to at least have some information, as guidance for participant or the participating company.’ [P3]*
Questions regarding added value of real-world data	*‘And what I expect, or what I’m a little bit afraid of, is that during that assessment, after three years, we will come to the conclusion that in more cases, other data bring more weight into the assessment then the collected data in the DAP. And then you could wonder, did we collect the right data, or did we only collect data because we wanted to come to another agreement and we did not want to immediately, fully reimburse the product?’ [P4]*
Lack of trust	*‘That [re-assessment] should be a criteria to be set in advance. And we have not seen anything for this yet. So I think it should be clear for all stakeholders. If you start managed entry agreements, you need to be clear on the criteria and the outcomes by which you will be measured. And I think this is too much in the open.’ [P3]*
Difficulty prescribing product by physicians	*‘[…] it did take quite some time for the hospitals to be onboarded. And they needed to hand over data to the AVL [NKI’s hospital] of course. Some unclarity about the ordering of the drug, I think also in the beginning. But probably we didn’t hear everything to be honest, because that needed to be solved as much as possible by the AVL [NKI’s hospital].’ [P5]*

All participants indicated that a key value of the DAP programme is achieving earlier access for patients to these drugs awaiting reimbursement. This fulfils a high unmet medical need.

However, some concerns exist whether long-term patient access is ensured in all cases. Re-assessment could lead to a negative outcome, ceasing patient access. Moreover, after positive re-assessment, DAP participation could complicate price renegotiations with the payer’s negotiation team, leading to a possible halt in access. For instance, MAHs could debate a recoup of their financial investment into the DAP.

#### Mitigating uncertainty on cost-effectiveness

The majority of MAHs agreed that due to uncertainty in the clinical data, payers face difficulty in predicting real-world effectiveness. This is also linked to the fact that the drugs included in the DAP mostly have an EMA conditional market approval status, as seen in Table 3.

#### Clear timeframes and roles of stakeholders

Regarding the specific roles of the stakeholders involved, MAHs stated that this was clearly communicated and well defined. As for timeframes, contracts have a clear start and ending date, which can be prolonged if the required number of patients included is not met. However, one MAH argued the process to be incoherent, without any party taking the lead. This absence leads to practical issues and inefficient implementation.

Moreover, MAHs stated to be unbeknownst whether recruitment prolongment is needed until the second year of the study. Also, some MAHs stated that clinicians were not aware of the discontinuation of patient enrolment. This led to MAHs actively reaching out to clinicians in order to stop further patient enrolment. Regarding the managed exit and re-assessment phase, MAHs were uninformed in which manner these procedures would proceed and what the prospected role of the MAH would be during this phase.

#### Real-world effectiveness in the Netherlands

The health insurer argued that clinical trials by default include patients who do not correspond well with the type of patients expected to receive treatment.

Gathering data from real-world treatment would address these uncertainties and provide some idea about how the treatment performs outside controlled clinical settings. However, MAHs argued that due to the absence of a clear, well-communicated definition of the existing uncertainties, it is challenging to predict whether the RWD collected will effectively address them.

### Challenges of participating in the DAP programme

#### Financial costs of data collection

One of the issues raised was the data management fee paid per patient for preparing the study initiation, collecting data from participating hospitals based on an electronic case report form, monitoring data, providing information to the participating centres and analysing and reporting the data. All MAHs believed that the data management fee was high compared with conventional data collection costs. Unanimously, participants stated it was difficult to obtain clearance within their global company structure for the extra fees of data collection in the Netherlands, as questions were raised regarding the details of the gathered data and what it would yield. Failing to achieve this fair market value assessment almost prohibited DAP participation. In their defence, the DAP’s governance committee argues that whereas the study population is small, the infrastructure and personnel costs are spread over a limited number of patients and hence do not offer a valid comparison with conventional data collection costs. Moreover, funding of the data collection could lead to a positive reimbursement decision and thus a return of investment for MAHs.

#### Rigidity of the financial OB-MEA arrangement

All MAHs argued that the fixed study protocol functions as an indirect selection criteria for products eligible for the DAP. For instance, the rigid cut-off period for the initial 16-week free-of-charge period can be skewed for certain products in terms of costs. For instance, some indications have short prognosis or treatment duration (e.g. gene therapy), or some products have high initial dosing followed by low maintenance doses. Therefore, the characteristics of the scheme itself can be seen as a selection criterion, prohibiting products from entering the programme.

#### Lack of transparency

All MAHs stated that some aspects of the DAP process were unclear to them or poorly communicated. Firstly, eligibility criteria were stated to be not established, changing over time and depending on the person asked. It is unclear how the payer defines ‘high unmet medical need’ for the products’ indications. Moreover, MAHs were not aware if all products receiving market approval based on non-randomised studies are eligible, or whether products authorised on the basis of randomised trials can also be enrolled. The payer argues that these products are permitted for enrolment as well, even as off-label products. Also, MAHs argue it is unknown whether products pending phase III or prolonged phase II results are eligible for the DAP. Furthermore, some MAHs argued that uncertainty in costs and financial motivations are key in the selection of products, which does not emerge from the selection criteria. Likewise, they question whether healthcare budget impact influences the selection of products.

As for ‘high unmet medical need’, the payer formulated this criterium as a situation where no alternative treatment for the patient population indicated exists (anymore). The payer remarks that for products to be eligible, a minimum level of the PASKWIL-NRS criteria should be met (Table 1). This means sufficient grounds exist to condone the lack of randomisation, for example, the rarity of an indication. There has been a case where the Dutch Medical Oncology Association was unable to assess the product as the indication was not rare enough. If a product has an ongoing RCT study, there is generally no DAP eligibility, and these results should be awaited to apply for reimbursement. Products authorised based on a non-randomised study could receive reimbursement outside of DAP, provided there are no uncertainties regarding their effectiveness or the uncertainties are addressed in another way.

For DAP, price is not a selection criterium but becomes part of the financial scheme when a drug is included.

Secondly, most MAHs stated that the accrual rate remained unknown to them for a long time during the implementation phase. Also, they were unsure why there was no power calculation conducted to assess for non-inferiority. The payer argues that it is difficult to predict how many patients are expected to enrol during the initial 2 years. The consideration of prolonging the patient recruitment phase can only be undertaken after 2 years of data collection or after the readout of the data cut off after 2.5 to 3 years of the start of implementation. Current competition laws limit contract terms between health insurers and the industry to 3 years.

Thirdly, some MAHs were unsure which specific criteria are used for (re-)assessment and what data are included in the assessment. Moreover, it is unknown how uncertainty regarding (real-world) effectiveness is defined, and consequently, how RWD will solve this uncertainty. The payer states that for re-assessment, PASKWIL-NRS criteria are used, and it is assessed whether the RWD is statistically different from the results of the pivotal trial. The final decision is based on all published data available at that time, for example, other RWE and clinical study reports.

In addition, one company mentioned that the trial organisation could have been more structured, with more active communication towards caregivers and hospital pharmacies. They also noted that support from the DAP’s governance committee or medical societies in aligning the different stakeholders would have been appreciated, as for example one hospital pharmacy refused to collaborate with the company.

#### Data sharing

Data sharing between the different parties involved, such as the Dutch National Cancer Institute and the payer, was mentioned as a difficult point. In addition, MAHs stated the need for information on required number of patients enrolled for both the test phase (first 16 weeks) and the commercial phase of the programme. One explanation for the issue around data sharing could be linked to the fact that there is no contract between the Dutch Cancer Institute and the payer, another reason being that clinicians in partaking hospitals do not always know who to reach out to concerning questions about the DAP programme.

#### Questions regarding added value of real-world data

MAHs unanimously state their uncertainty as to what extent the RWE will support reimbursement decisions. This uncertainty is linked to the unknown definition of uncertainty and untransparent eligibility criteria. Some MAHs questioned whether their study population was that different from the Dutch patients expected to receive the treatment, and whether gathering RWE is therefore necessary. Also, some MAHs argued that additional data from phase II/IVI trials, whether or not required by conditional market approval, are more valuable for long-term access to these products. The payer confirms that in one case, a DAP-enrolled product received reimbursement after new data publications became available, wherefore in retrospect, the DAP programme had not been needed. Consequently, these products should not be eligible for the DAP.

#### Lack of trust

An overarching theme that emerged from the interviews is the MAHs’ lack of trust in stakeholders (payers and hospitals) and the value of the DAP programme. This challenge is linked to issues in both communication and transparency between stakeholders. Firstly, MAHs resent the lack of a clear definition of uncertainty and the absence of transparent eligibility and re-assessment criteria. MAHs argue that without knowing the origin of uncertainty, they find it difficulty predicting whether the gathered RWD will solve this uncertainty. In the view of MAHs, the data collection aspect of the DAP lacks a clearly defined clinical rationale. Moreover, one MAH stated that the payer can change the DAP’s conditions during the implementation phase without consultation with MAHs, as the payer views the field as dynamic.

Secondly, according to some MAHs, the data collection fee is much higher than the typical costs of data collection, rendering suspicion of the fee being a revenue source for hospitals. Moreover, MAHs reckon that financial motivations originating from the payer are factored into the selection of these products.

Thirdly, one MAH pointed out that there is a risk of conflict of interest regarding the double role of certain committee members. Both the selection and exit of products are determined together by members of the DAP’s governance committee and members of the Dutch Association for Medical Oncology . In addition, individuals can have occupations in multiple committees, granting these individuals a key role in the programme.

From payer’s perspective, trust issues would be mitigated if there would be a better understanding of why the current landscape in the assessment of these products calls for a solution, which the DAP may provide. The underlying idea is that MAHs, payers and the medical society are trying to address the evidence gap. While traditionally, it is the responsibility of the MAH to provide robust evidence in the form of two randomised trials. The payer also hopes that examples of products receiving full reimbursement after participating in DAP, peer-reviewed publications, cohort reports and the annual meeting organised by the DAP team – to inform companies and address their questions and challenges – would improve trust in the programme.

#### Difficulty prescribing product by physicians

Several practical challenges surfaced during the implementation phase. The payer usually select specific hospitals with high expertise in treating the indication. MAHs discuss a contract with these hospital on terms of supplying and pricing. The hospitals participating are limited to a selected number of expert centres. Physicians at non-expert centres are not always aware of the existence of this treatment option, leading to lower referrals towards expert centres.

Also, as for the ease of prescribing a product within the DAP for clinicians, there was a clear discrepancy between MAHs. While some MAHs stated clinicians were comfortable using the product within DAP, in contrast, other MAHs argued it was a time-consuming process and an administrative burden for physicians. Furthermore, one MAH stated that physicians did not know how to reach out to the DAP committee or management team and therefore instead opted to reach out to MAHs.

Also, one MAH stated that for their product, some patients had the drug prescribed but were not included in the study. Physicians should send each patient for review to the Dutch Cancer Institute in order to have them enrolled in the study. The payer lacks the ability to monitor for this discrepancy as there is no data exchange in between due to the absence of a contract between the payer and the Dutch Cancer Institute. This discrepancy results in study selection bias and difficulty in the implementation of the financial agreement. [Explanation by DAP-management team: All serious protocol deviations, including the rare cases in which a patient has been mistakenly placed on the medication outside of the DAP, are discussed with the payer of the patient to determine whether the costs can be covered from the healthcare budget or whether a compassionate use request should be submitted to the MAH. A contract between the payer and the Dutch Cancer Institute is legally not allowed due to privacy reasons.]

## Discussion

This qualitative study gives insights into the complicated processes of implementing an innovative, clinician-led early access scheme linking evidence generation and reimbursement for tumour-agnostic cancer medicines in the Netherlands. The analysis of the interviews of all involved stakeholders made it clear that while all stakeholders acknowledged several enablers, many challenges still remain for a smooth implementation of the programme. Facilitating patient access and potentially achieving regular reimbursement are key reasons for participating in the programme. However, concerns regarding the lack of clarity, challenges in data sharing and a limited trust can affect the feasibility of the DAP and the willingness of MAHs to participate. Alignment and permission from the corporate headquarters of the MAH seemed to be one of the major challenges for the local branch of the MAH to justify the participation in the DAP.

The study findings align with previous assessments of the implementation of OB-MEAs. Several publications address difficulties in generating evidence, high administrative burdens and time-consuming negotiations [8–10, 26, 27]. Due to this complexity, healthcare decision makers are driven to opt for simpler financial MEAs (e.g. discounts) instead. The requirements needed to make MEAs feasible are well discussed in literature. Among these, a strong alignment of objectives between health decision makers and MAHs is stated [10]. Arguably, in the DAP programme, this alignment could be improved, as highlighted from the identified challenges. As MAHs commit to a long-term agreement that involves high financial (data management fee and free-of-charge clawbacks) and implementation costs (e.g. negotiation time), it is crucial to have upfront guidance on the managed exit or re-assessment phase.

While it has to be acknowledged that DAP provides non-randomised data, the added value of DAP-like registries is that they can reduce health system’s uncertainty regarding how the treatment performs in broader, unselected populations, thereby addressing population bias. An additional advantage is that these data can later help payers and companies to make more informed financial arrangements. Representatives of the DAP management argue that concerns about an anticipated unchecked rise in healthcare budget costs make healthcare payers and the Dutch HTA body cautious. This can make the DAP an important decision-making instrument in case of unclear reimbursement recommendations.

The findings of this study have great learning potentials for other countries. Literature suggests that insights into the experiences and interests of stakeholders can be essential in understanding the feasibility of successfully implementing MEA (like-) programmes [27, 28, 29, 30]. This may give healthcare decision-makers the necessary tools to improve these types of agreements and therefore ensure that expenditures of the healthcare system are used efficiently and effectively while also ensuring patient access to these innovative therapies.

However, alike their pivotal trials, RWD gathering faces challenges due to small patient numbers. In multiple cases, the DAP’s recruitment phase had to be prolonged in order to collect sufficient data. Implementing the DAP programme in other EU countries could facilitate access and support the joint generation of RWD. A similar programme, the Drug Rediscovery Protocol (DRUP), which is aimed to improve patient access to off-label oncology treatments, makes use of such a collaboration [31]. This collaboration is incorporated in the PRIME-ROSE (Precision Cancer Medicine Repurposing System Using Pragmatic Clinical Trials) consortium, which aggregates data from DRUP-like studies conducted in multiple countries [32, 33]. A similar initiative for DAP-like studies may significantly improve recruitment rates and accelerate evidence building [9, 34, 35, 36, 37].

In a recent evaluation of the Dutch HTA body’s coverage with evidence programme as well as the DAP programme, future considerations were mentioned including the consideration of harmonising all different OB-MEA systems in the Netherlands under the umbrella of one. Another point raised was to also adjust the processes to also include diagnostic tests in the reimbursement process. This originated from the Larotrectinib/Entrectinib case, where the drug was reimbursed after inclusion in the DAP, but the path for the molecular diagnostics at that time was unclear, leading to a low rate of diagnosed patients. Overall, MAHs found the DAP process as more transparent and a quicker process than the other Dutch OB-MEA process by the Dutch HTA body.

While we have to acknowledge that generalisability is the main limitation of this study, we believe that this is at the same time also a strength of the study. We use the DAP protocol from the Netherlands as a case study to describe challenges in implementing early access schemes, which could vastly differ between high- and low-income countries or between single- or multi-payer systems [9]. Also, the Netherlands possesses a good research infrastructure and strong research institutions like the Netherlands Cancer Institute. Fragmentation of healthcare structure can make undertaking MEAs in practice more difficult [10]. However, we believe this case report provides valuable insights from all involved stakeholders on how to practically implement a clinician-led early access scheme, which provides the opportunity for other countries to learn from.

## Conclusion

In conclusion, coverage with evidence development programmes like the DAP may be a promising solution to mitigate uncertainty for healthcare payers and simultaneously provide patient access to innovative therapies. Such bottom-up approaches are valuable because they draw on the practical insights of clinicians and payers, aligning access decisions with RWE to ensure they are both practical and sustainable.

## Supplementary Material



## Data Availability

This study is based information obtained through interviews. Due to privacy protection, the data of the interviews cannot be made publicly available.
