# Supplementary material for: Stakeholders’ experiences with a clinician-led access programme linking evidence generation and reimbursement for precision cancer treatments: the drug access protocol in the Netherlands
Source: Acta Oncol. 2026 Jan 14;65:45000. doi: 10.2340/1651-226X.2026.45000 (PMC12816994; doi:10.2340/1651-226X.2026.45000)
Supplement: Supplementary file 1 [file AO-65-45000-s1.pdf]

Supplementary material has been published as submitted. It has not been copyedited, or typeset by Acta Oncologica

Supplement material – Interview Guide

Interview guide "*Experiences of the pharmaceutical industry with the Dutch Drug Access Protocol*"

Introduction to the interview

We thank you for your time and willingness to share your expertise with us today. As explained in our invitation, we are interested in learning about the experiences and perspectives of the pharmaceutical industry and patient advocacy groups with the Dutch Drug Access Protocol (DAP).

The overall goal of our interview is to summarize experiences of the industry with the DAP.

This interview will not take longer than 60 minutes. To ensure accuracy of our summary of responses from each interview, we would like to audio-record our conversation. This recording will allow us to review and correctly summarize the information that you mention. Information from the interviews will be summarized without identifying interviewees.

Some responses may make important points, which we may want to highlight specifically but we will **not** make any references to your person. Do we have your permission to proceed with the interview and audio record this conversation?

- ❑ Yes, continue with audio
- ❑ No, continue without audio (it may take us a bit longer to take notes during the call.)

Do you have any questions before we proceed?

Overview of the interview

We greatly appreciate if you could respond using examples and share your experiences of what may have worked well and what may not have worked prior, during and after the DAP protocol. For this purpose, we have structured the interview guide into four sections:

1. The Drug Access Protocol process
2. Data collection and protocol agreement
3. Financial agreement
4. Open reflections

The Drug Access Protocol process

In the attached file, we have displayed the DAP process from a health system perspective. We are now interested to learn from you the following points:

1. Your company has been selected for this interview due to your previous or current collaboration in DAP. How do you think that DAP has contributed to the availability of your product?
2. Do you agree with the way the DAP process is displayed? What changes would you propose in this flowchart?
3. Do you think the process is transparent enough?
4. Do you think the eligibility criteria for entry in the DAP protocol are clear?
5. What were the delaying factors in starting with DAP?
6. Do you think that DAP should be rolled-out to other EU countries? What would be the advantages and disadvantages?

Data collection period

In this section, we are specifically interested to learn from your experience in collecting data.

1. Do you think the selected endpoints and follow-up periods are appropriate (feasible and clinical relevant) for gathering the required data?
2. What type of data did you receive during the DAP period regarding your product?
3. What type of data would be useful to you (and why?)?
4. Were there requests to make drug available for patients who did not meet de eligibility criteria of DAP? Did you provide access?
5. What worked well/didn't work well during the data collection period?

Financial agreement

One might argue, that the DAP can be considered as an outcome-based managed entry agreement. We are now interested in your experience with the financial agreement of the DAP.

1. Do you consider the DAP as an outcome-based Managed Entry Agreements? If so, what MEA elements were part of this?
2. Do you think that this is a pragmatic programme? Please elaborate.
3. How evident are the financial implications at the time of establishing the financial agreement?
4. Do you think implementation costs inhibits selecting more complicated forms of MEA's?

Open reflection

1. Do you think the DAP is successful in achieving its goals in terms of improving patient access and reimbursement decisions? Why/why not?
2. Based on your experience, what would you continue to do/do differently in the future? Why?
